# Application of the Born Model to Describe Salt Partitioning in Hydrated Polymers

**DOI:** 10.1021/acsmacrolett.4c00048

**Published:** 2024-04-16

**Authors:** Sean M. Bannon, Geoffrey M. Geise

**Affiliations:** Department of Chemical Engineering, University of Virginia, 385 McCormick Road, Charlottesville, Virginia 22903, United States

## Abstract

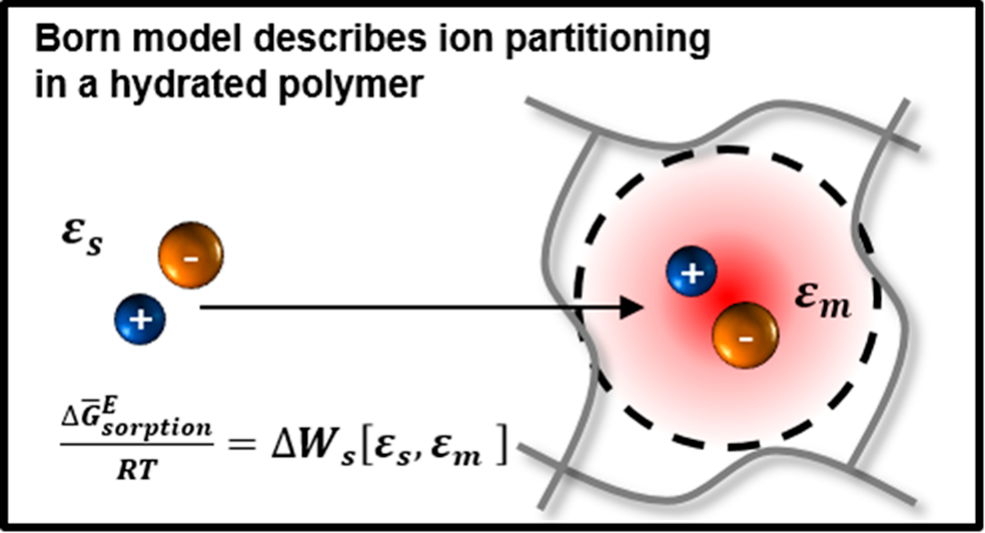

The classic Born model can be used to predict salt partitioning properties observed in hydrated polymers, but there are often significant quantitative discrepancies between these predictions and the experimental data. Here, we use an updated version of the Born model, reformulated to account for the local environment and mesh size of a hydrated polymer, to describe previously published NaCl, KCl, and LiCl partitioning properties of model cross-linked poly(ethylene glycol) diacrylate polymers. This reformulated Born model describes the influence of polymer structure (i.e., network mesh size and its relationship with water content) and external salt concentration on salt partitioning in the polymers with a significant improvement relative to the classic Born model. The updated model most effectively describes NaCl partitioning properties and provides an additional fundamental understanding of salt partitioning processes, for NaCl, KCl, and LiCl, in hydrated polymers that are of interest for a variety of environmental and biological applications.

Many processes that are relevant for environmental and biological applications (e.g., separation^1−3^ and drug delivery^4−6^ processes) require equilibration of a hydrated polymer with an aqueous electrolyte solution. Under these circumstances, to minimize the free energy between the polymer and solution, mobile salt (i.e., ions) partitions from the aqueous electrolyte into the hydrated polymer.^2,7^ This salt partitioning process is governed by nonideal thermodynamic interactions between ions, water molecules, and polymer. As such, specific details of the polymer structure have a significant impact on salt partitioning. Understanding how polymer chemistry influences these thermodynamic interactions would provide a fundamental understanding of the salt partitioning phenomenon in many hydrated polymer-based processes.

Modeling approaches that quantify thermodynamic interactions in solution and polymer can be used to probe the relationship between polymer chemistry and polymer salt partitioning properties. In the simplest case, these thermodynamic interactions can be quantified using the classic Born model,^8^ which describes interactions between ions and their induced polarization charges (which are commonly referred to as self-interactions or ion solvation interactions).^9^ Previous reports, however, have found significant quantitative discrepancies between classic Born model predictions and experimental data.^10−13^ There have been significant efforts to reconcile these discrepancies between classic Born model predictions and salt partitioning data in hydrated polymers,^9−17^ and currently, it is generally accepted that the Born model may be too simple to fully describe these salt partitioning processes.^9^

In this Letter, we propose an updated modeling approach based on the classic Born model that describes salt partitioning in uncharged hydrated polymers as a function of their water content. This model is developed by accounting for the hydrated polymer mesh size in an updated version of the Born model and by using the Maxwell Garnett model to describe the hydration dependent dielectric constant of the polymer. This updated modeling approach provides remarkably accurate descriptions of previously reported salt partitioning properties in a series of uncharged poly(ethylene glycol) diacrylate (XLPEGDA) polymers over a range of water volume fractions of 0.2–0.80^18^ when equilibrated with 0.01–1 M NaCl.^19^ For hydrated polymers equilibrated with 0.01–1 M LiCl or KCl,^19^ model predictions are less accurate. These results suggest that the Born model can accurately describe simple salt partitioning processes in hydrated uncharged polymers, which provides a fundamental understanding of how polymer chemistry influences salt partitioning processes.

Subsequently, we outline the modeling approaches used to describe salt partitioning in hydrated polymers calculated using either the classic Born model or our updated Born model (referred to herein as the Freger–Born model). The subscript 0 is provided to differentiate values predicted using the classic Born model from those predicted using the Freger-Born model that will be differentiated subsequently with subscript 1. Key equations are provided here, and additional information regarding their derivation is provided in Section S1.

Salt partitioning processes are characterized by the hydrated polymer salt partition (or sorption) coefficient, *K*_s_, which, for a 1–1 electrolyte (e.g., NaCl), is defined as^11,12,20^
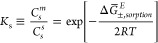
1where *C*_s_^m^ and *C*_s_^s^ are the equilibrium concentrations of mobile salt in the polymer and external solution, respectively, *R* is the gas constant, *T* is the temperature, and Δ*G̅*_±,sorption_^*E*^ is the difference (between polymer and solution) in the mean ionic partial molar excess Gibbs free energy associated with the partitioning process (Section S1.1). In the simplest case, [Δ*G̅*_±,sorption_^*E*^/*RT*] is related to measurable quantities via a mean ionic excess solvation energy (eq S7) that accounts for the difference (between polymer and solution) in ion solvation interactions.^9,14,21^ This mean ionic excess solvation energy, Δ*W*_s__,0_, can be calculated using the classic Born model:^9^
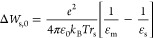
2where *e* is the elementary charge, ε_0_ is the vacuum permittivity, *k*_B_ is the Boltzmann constant, ε_m_ and ε_s_ are the dielectric constants of the polymer and solution, respectively, and *r*_s_ is the mean ionic cavity radius for the salt, which is defined in eq S10 (for NaCl, *r*_s_ = 1.87 Å). It is important to note that eq 2 is a specific application of the Born model that describes the mean ionic excess solvation energy for a 1–1 electrolyte, and a more general form is provided in the Supporting Information (eq S9). The Born model is useful because, given the polymer dielectric constant, it can be used to quantitatively describe the energy associated with salt partitioning processes in hydrated polymers.^9−11,22^ Effectively, the model suggests that as the polymer dielectric constant increases, the excess solvation energy reduces (eq 2; i.e., self-interactions become increasingly thermodynamically favorable in the polymer), and as a result, the salt partition coefficient increases.

Experimental structure/property relationships of hydrated polymers suggest that, generally, the dielectric constant of a hydrated polymer increases with increasing polymer water content.^10,22−25^ This relationship can be quantitatively described using models, such as the Maxwell Garnett model, to describe the dielectric constant of a heterogeneous material with one phase (phase 2) dispersed throughout another continuous phase (phase 1), as^26^
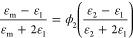
3where ε_1_ and ε_2_ are the dielectric constants of the pure continuous and disperse phases, respectively, and ϕ_2_ is the volume fraction of the disperse phase in the material. In many hydrated polymers, the Maxwell Garnett model most accurately describes the polymer dielectric constant when the polymer is taken as the continuous phase (Section S2).^26^ Here, we assume that a polymer-continuous application of the Maxwell Garnett model describes the dielectric constant of all polymers considered in this investigation (Section S2).

Combining the definition of the salt partition coefficient with the Born and Maxwell Garnett models (eqs 1, 2, and 3, respectively) results in a so-called Electrostatic Theory that can describe how any modification of water content should influence the salt partition coefficient in a hydrated polymer.^11^ Previously, Electrostatic Theory has successfully been used to qualitatively describe the relationship between polymer water volume fraction and salt partitioning data.^10,11,27,28^ For example, in model cross-linked poly(ethylene glycol) diacrylate hydrogels (XLPEGDA), NaCl partition coefficients increase with increasing polymer water volume fraction (Figure 1).^18,19^ This result is qualitatively consistent with Born model predictions (Figure 1) because the polymer dielectric constant increases with an increased water volume fraction (eq 3). However, as discussed previously, quantitative discrepancies between classic Born model predictions using this framework and experimental data are generally large (e.g., approximately an order of magnitude; Figure 1).^9,10^

**Figure 1:**
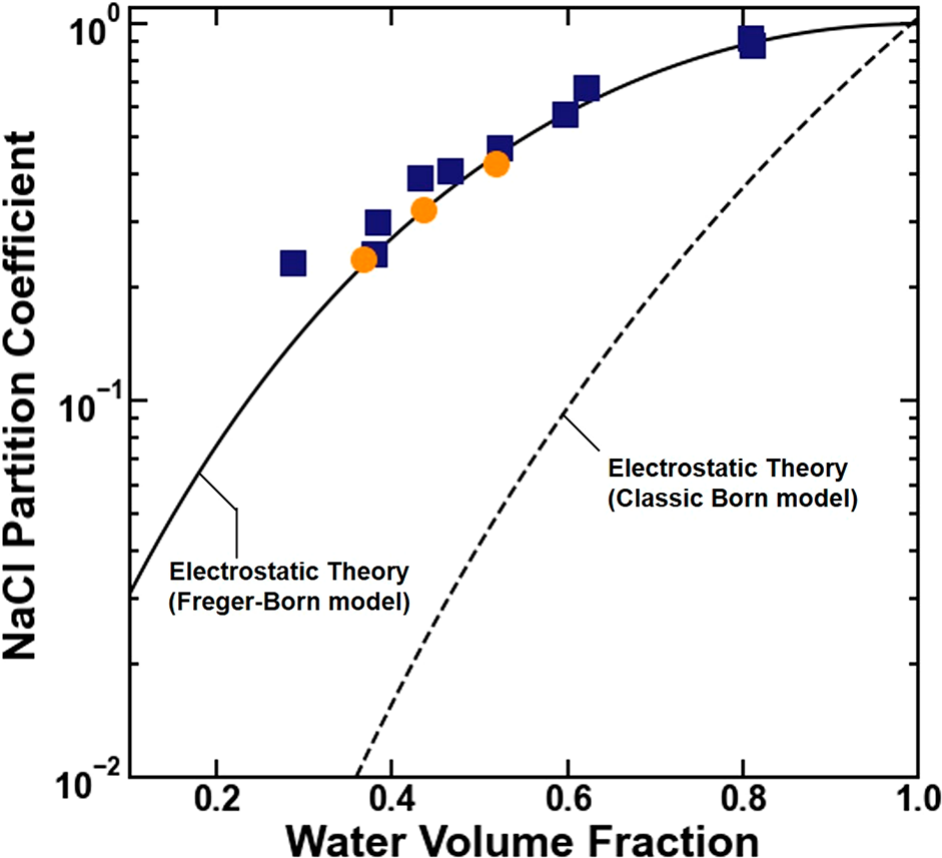
NaCl partition coefficient data in units of [(mol/L (water sorbed))/(mol/L (solution))]^29^ plotted as a function of water volume fraction for XLPEGDA polymers equilibrated with 0.1 M NaCl as characterized by Ju et al. (■)^18^ and Jang et al. (●).^19^ The dashed line represents model predictions of the Electrostatic Theory with the classic Born model (eqs 1, 2, and 3), whereas the solid line represents model predictions of the Electrostatic Theory with the Freger–Born model (eqs 1, 3, and 4).

One explanation is that these discrepancies are observed because the classic Born model was derived to describe thermodynamic interactions in homogeneous dielectric continua (i.e., materials of uniform dielectric constant).^8−10^ At sufficiently small length scales, hydrated polymers contain distinct water-rich and polymer-rich regions, and the dielectric constant in these regions is less well-defined and/or likely nonuniform.^9^ Such interfaces between water- and polymer-rich regions within a polymer could significantly influence the excess solvation energy if ions experience a discontinuity in the effective dielectric constant near or at these interfaces.

Recently, Freger proposed a theoretical modification of the Born model to describe the influence of these interfaces on the excess solvation energy.^9^ In this Freger–Born model, for the limiting case where the dielectric constant of the water-rich region is approximated as that of the external solution, the mean ionic excess solvation energy, Δ*W*_s__,1_, is^9,14^
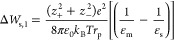
4where *z*_+_ and *z*_–_ are the valences of the cation and anion, respectively, and *r*_p_ is a characteristic hydrated void space within the polymer. Additional details regarding the derivation of eq 4 are provided in the Supporting Information.

To the authors’ knowledge there has yet to be an application of Freger’s reformulation of the Born model (eq 4) to describe experimentally measured salt partitioning in hydrated polymers. Combining the definition of the salt partition coefficient with the Freger–Born model and the Maxwell Garnett model (eqs 1, 2, and 4) result in an updated Electrostatic Theory that accounts for the influence of polymer microstructure, through the characteristic void space, on salt partitioning. Here, we take this characteristic void space as half the polymer mesh size (i.e., 2*r*_p_ = ζ), which can be estimated from experimental data (Section S3).^19,30^

The Electrostatic Theory calculated using the Freger–Born model predicts that the NaCl partition coefficients increase with increasing water volume fraction because the polymer mesh size and dielectric constant increase with increasing water volume fraction (Figure 1). A theoretical limitation of the Freger–Born model is that it is mathematically valid for the case where the hydrated polymer mesh size is reasonably larger than the mean ionic radius (i.e., *r*_p_ > *r*_i_).^9^ Because the hydrated polymer mesh size is proportional to the water-volume fraction, this condition is tested in lower water content XLPEGDA (Figure 1). Visual agreement between the model predictions and experimental data does not change in low water content XLPEGDA (Figure 1), suggesting that for all the materials studied here, the hydrated mesh size is sufficiently larger than the mean ionic radius for the Freger–Born model to be valid.

The quantitative agreement between experimental data and model predictions, calculated using the Electrostatic Theory with the Freger-Born model (eqs 1, 3, and 4), significantly improves (as can be seen visually in Figure 1) compared to the situation with the model predictions calculated using the Electrostatic Theory with the classic Born model (eqs 1, 2, and 3). This improvement corresponded to root-mean-square (RMS) log errors^31^ of 0.08 and 1.14 for the Freger–Born and classic Born models, respectively. These results suggest that, to a first approximation, accounting for the polymer mesh size when calculating the excess solvation energy significantly improves the accuracy of the Electrostatic Theory.

The Electrostatic Theory with the Freger–Born model may also be useful to describe the influence of the external salt concentration on the salt partitioning properties of hydrated polymers. Generally, the dielectric constant of aqueous electrolyte solutions decreases with increasing concentration, and this concentration-dependent dielectric constant can be quantitatively modeled using experimental correlation models (Section S4).^32,33^ For example, for NaCl, the dielectric constant is calculated as^33^
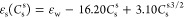
5where *ε*_w_ is the dielectric constant of pure water; note that the units of *C*_s_^s^ in eq 5 are in [mol/L]. While experimental results that characterize the influence of dielectric constant of hydrated polymers are limited, available results generally suggest that the dielectric constant of a hydrated polymer is less dependent on external salt concentration than the dielectric constant of an aqueous electrolyte solution.^14,26^ Therefore, to model the influence of external salt concentration on salt partitioning, ε_m_ can be treated as a constant (estimated as its value in a polymer equilibrated with deionized water), and ε_s_ can be calculated using eq 5.

Jang et al. characterized the influence of external salt concentration on NaCl partitioning in the same series of XLPEGDA hydrogels (where the polymer nomenclature is such that the relative order of polymer water volume fraction was XL40 > XL20 > and XL0; Figure 2).^19^ They observed that the NaCl partition coefficient increased with increasing external solution concentration (Figure 2). The Born model qualitatively describes this phenomenon because the excess solvation energy decreases as the solution dielectric constant increases (eqs 2 and 4). Additionally, the predictions of the Electrostatic Theory with the Freger–Born model have striking visual agreement with the experimental data (Figure 2). The RMS log errors for these data, determined for the XL0, XL20, and XL40 polymers over the full range of external salt concentrations, were 0.05, 0.02, and 0.07, respectively. These results suggest that the Electrostatic Theory with the Freger–Born model can be used to describe the influence of the external salt solution concentration on salt partitioning in hydrated polymers.

**Figure 2:**
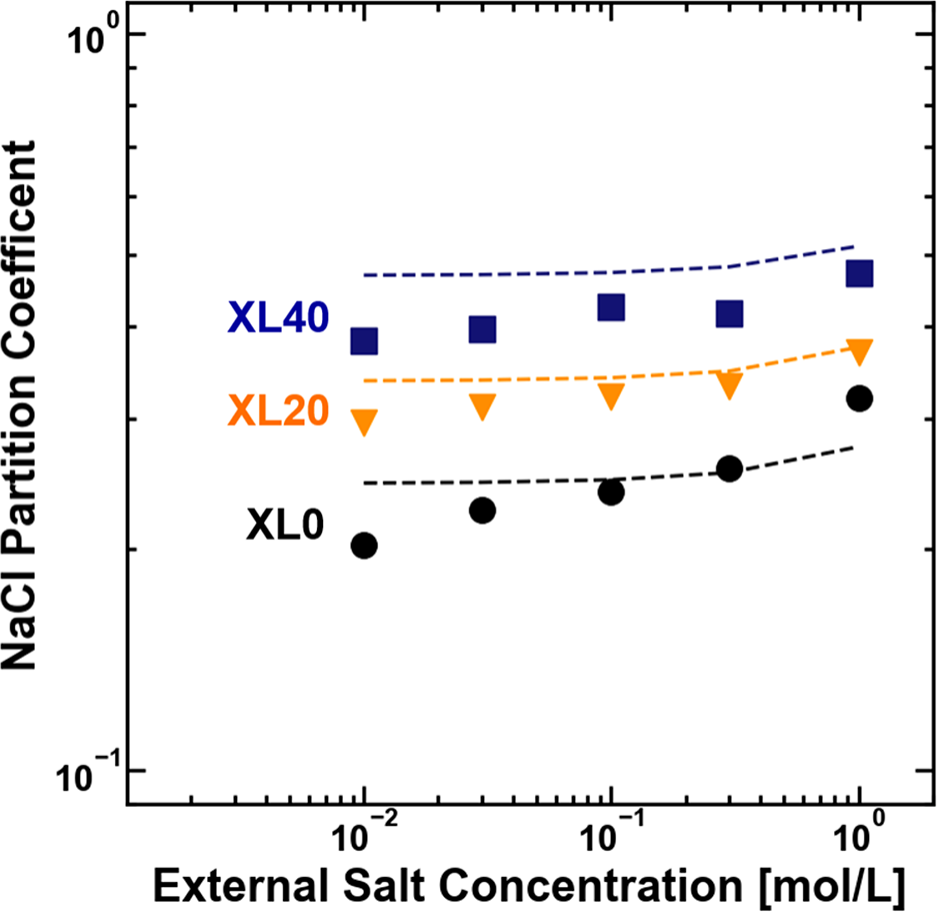
NaCl partition coefficient data in units of [(mol/L (water sorbed))/(mol/L (solution))]^29^ for XL0 (●), XL20 (▼), and XL40 (■) reported as a function of external salt concentration.^19^ Dashed lines represent model predictions of the electrostatic with the Freger–Born model (eqs 1, 3, and 4).

Lastly, a considerable advantage of the Electrostatic Theory with the classic Born model is that theoretically, it is salt specific (i.e., describes the influence of salt, or ion, identity on salt partitioning) because the classic Born model is dependent on *r*_s_ (eq 2). The limiting case described by the Freger-Born model causes the dependence on *r*_s_ to vanish, which results in a loss of salt specificity (i.e., suggests that salt partitioning in the polymer is dependent only on ion valence; eq 4). Currently, it is poorly understood if the salt-specific partitioning properties of hydrated polymers are qualitatively described by classic Born predictions. As a result, is unclear if the loss of salt specificity in the Freger–Born model is significant.

To test the ability of the Electrostatic Theory to describe salt-specific partitioning properties of hydrated polymers, we applied the model to KCl and LiCl partitioning data characterized for XLPEGDA by Jang et al.^19^ For reference, the mean ionic cavity radii for KCl and LiCl are 2.11 and 1.61 Å, respectively (calculated via eq S10).^34^ Jang et al. observed that the relative order of salt partition coefficients was *K*_KCl_ > *K*_LiCl_ > *K*_NaCl_ (Figure 3).^19^ This result is not qualitatively consistent with predictions of the Electrostatic Theory calculated using the classic Born model, which suggest that salt partitioning should increase with increasing mean ionic cavity radii (Figure 3). Additionally, the Electrostatic Theory with the Freger–Born model describes LiCl, KCl, and NaCl partitioning in the XLPEGDA polymers with significantly improved quantitative accuracy relative to the classic Born model (Figure 3 and Table S3). For this reason, the loss of salt specificity in the Freger–Born model does not reduce the agreement between the model predictions and experimental data.

**Figure 3:**
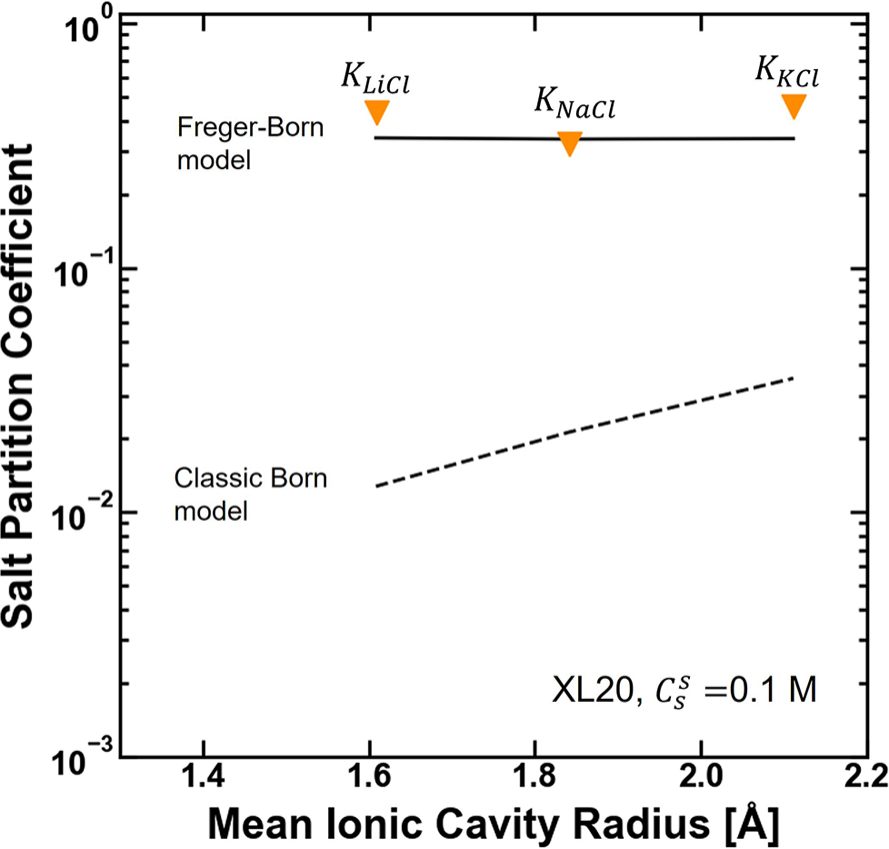
KCl, LiCl, and NaCl partition coefficients [(mol/L (water sorbed))/(mol/L (solution))]^29^ reported as a function of the mean ionic cavity radii for XL20 equilibrated with 0.1 M NaCl, LiCl, or KCl. The mean ionic cavity radii were calculated from the ionic cavity radii as reported by Duignan et al.^34^ via eq S10. The dashed line represents model predictions of the Electrostatic Theory with the classic Born model (eqs 1, 2, and 3), whereas the solid line represents model predictions of the Electrostatic Theory with the Freger–Born model (eqs 1, 3, and 4).

A possible explanation for this result is that other thermodynamic interactions besides the self-interactions described by the Born model, (e.g., ion association interactions or long-range electrostatic interactions) may influence KCl and LiCl partitioning properties in XLPEGDA to a greater extent than NaCl partitioning properties. This explanation is consistent with a recent molecular dynamics simulation investigation of the XLPEGDA polymers, where association interactions between potassium ions and ethylene oxide repeat units along the polymer backbone were generally more favorable than sodium/ethylene oxide interactions or lithium/ethylene oxide interactions.^35^ Similarly, association interactions between lithium and water molecules are generally more thermodynamically favorable than those between sodium and water molecules or potassium and water molecules.^19^ These results suggest that to accurately describe ion-specific partition data, it may be necessary to derive mathematical models that account for the influence of secondary interactions (i.e., ion association) on salt partitioning.

Overall, the Electrostatic Theory calculated using the Freger-Born model significantly improves upon the ability of the Electrostatic Theory calculated using the classic Born model to describe salt partitioning in XLPEGDA and highlights the influence of the network mesh size on salt partitioning. Additionally, the new approach accurately describes the influence of external salt concentration on the salt partitioning properties of hydrated XLPEGDA. Finally, we found that neither version of the Electrostatic Theory fully describes the nuances of specific salt (i.e., KCl or LiCl) partitioning in XLPEGDA. These results may be useful to provide a fundamental understanding of the salt partitioning properties of hydrated polymers and provide an analytical technique to predict the influence of polymer properties (i.e., mesh size and/or water content) and external conditions (i.e., salt concentration) on salt partitioning in hydrated polymers.

## Experimental Section

All data used in this investigation were reported previously for XLPEGDA polymers prepared via a UV cross-linking procedure.^19,30^ All parameters used in the models (i.e., mesh size and dielectric constant) were determined using data for polymers equilibrated with deionized water. For convenience, these parameters for the XL0, XL20, and XL40 polymers are listed in Table 1.

**Table 1:** Polymer Properties and Model Parameters for XL0, XL20, and XL40

polymer	ζ (Å)	ε_m_
XL0	12	23
XL20	13	27
XL40	15	31

## Supporting Information Available

Model derivation, Maxwell Garnett model, mesh size calculation, empirical correlation models for dielectric constant of electrolyte solutions, additional model applications, and error (PDF)
