# Supplementary material for: Application of the Born Model to Describe Salt Partitioning in Hydrated Polymers
Source: ACS Macro Lett. 2024 Apr 16;13(5):515–20. doi: 10.1021/acsmacrolett.4c00048 (PMC11112736; doi:10.1021/acsmacrolett.4c00048)
Supplement: Supplementary file 1 — mz4c00048_si_001.pdf [file mz4c00048_si_001.pdf]

<u>Supporting Information</u>

Application of the Born model to describe salt partitioning in hydrated polymers

*Sean M. Bannon and Geoffrey M. Geise**

Department of Chemical Engineering

University of Virginia

385 McCormick Road

Charlottesville, Virginia 22903

*To whom correspondence should be addressed: geise@virginia.edu

(Tel: +1-434-924-6248, Fax: +1-434-982-2658)

Section S1. Derivation of the Electrostatic Theory

Section S1.1. Equilibrium condition for salt partitioning

The equilibrium condition for a hydrated polymer equilibrated with an aqueous electrolyte is:[1]

$$a_{\pm}^{m} = a_{\pm}^{s} \qquad \text{Eq. S1}$$

where $a_{\pm}^{m}$ and $a_{\pm}^{s}$ are the mean ionic activities in the polymer and external solution, respectively. The superscript m is used to denote the polymer (i.e., membrane) phase, and the superscript s is used to denote the solution phase. Rearranging Eq. S1 and expressing the mean ionic activity in terms of the mean ionic concentration, $C_{\pm}$, and the mean ionic activity coefficient, $\gamma_{\pm}$, yields:[1,2]

$$\frac{C_{\pm}^{m}}{C_{\pm}^{s}} = \frac{\gamma_{\pm}^{s}}{\gamma_{\pm}^{m}} \qquad \text{Eq. S2}$$

For a 1-1 electrolyte solution, the concentration of cations and anions in the solution must be equal to maintain electronuetrality, i.e., $C_{\pm}^{s} = C_{s}^{s}$ (where C_{s}^{s} defines the concentration of salt in the external solution).[3] Similarly, for an uncharged polymer equilibrated with a 1-1 electrolyte, $C_{\pm}^{m} = C_{s}^{m}$. Therefore, Eq. S2 can be written as:[4]

$$\frac{C_{s}^{m}}{C_{s}^{s}} = \frac{\gamma_{\pm}^{s}}{\gamma_{\pm}^{m}} \qquad \text{Eq. S2b}$$

The salt partition (or sorption) coefficient for a hydrated polymer equilibrated with an aqueous electrolyte, K_s, is defined as the ratio of salt in the polymer relative to that in the external solution (i.e., $K_s \equiv C_{s}^{m}/C_{s}^{s}$).[4] Therefore, the salt partition coefficient for an uncharged polymer equilibrated with a 1-1 electrolyte can be defined as:

$$K_s = \frac{\gamma_{\pm}^{s}}{\gamma_{\pm}^{m}} \qquad \text{Eq. S3}$$

For an aqueous 1-1 salt, the mean ionic activity coefficient is defined using the molar excess Gibbs free energy, ${\bar{G}_{\pm}}^{E}$, as:[2,5]

$$\gamma_{\pm}^{j} = \exp\left[\frac{{\bar{G}_{\pm}}^{E,j}}{2RT}\right] \qquad \text{Eq. S4}$$

where R and T are the gas constant and temperature, respectively, and j is an arbitrary index denoting the phase. Therefore, the ratio of activity coefficients in solution and polymer can be defined as:

$$\frac{\gamma_{\pm}^{s}}{\gamma_{\pm}^{m}} = \exp\left[\frac{{\bar{G}_{\pm}}^{E,s} - {\bar{G}_{\pm}}^{E,m}}{2RT}\right] = \exp\left[-\frac{\Delta\bar{G}_{\pm,sorption}^{E}}{2RT}\right] \qquad \text{Eq. S5}$$

where $\Delta\bar{G}_{\pm,sorption}^{E}$ is the change in partial molar excess Gibbs free energy associated with the salt partitioning process, which is defined as $\Delta\bar{G}_{\pm,sorption}^{E} = {\bar{G}_{\pm}}^{E,m} - {\bar{G}_{\pm}}^{E,s}$. Combining Eq. S5 with Eq. S3 results in the following definition of the salt sorption coefficient of an uncharged polymer equilibrated with a 1-1 electrolyte:

$$K_s = \exp\left[-\frac{\Delta\bar{G}_{\pm,sorption}^{E}}{2RT}\right] \qquad \text{Eq. S6}$$

Section S1.2. Excess solvation energy

Electrostatic Theory results from relating $\Delta\bar{G}_{\pm,sorption}^{E}$ to experimentally measurable quantities. One approach is to relate $\Delta\bar{G}_{\pm,sorption}^{E}$ to the mean ionic excess solvation energy as:

$$\frac{\Delta\bar{G}_{\pm,sorption}^{E}}{RT} = \Delta W_s \qquad \text{Eq. S7}$$

where ΔW_s is the mean ionic excess solvation energy (in units of $k_B T$). In this sub-section, we outline two approaches, herein referred to as the classic Born model and Freger-Born model, to evaluate the mean ionic excess solvation energy.

The classic Born model defines the excess solvation energy of ion i, $\Delta W_{i,0}$, as:[2,6,7]

$$\Delta W_{i,0} = \frac{z_i^2 e^2}{8\pi\varepsilon_0 k_B T r_i}\left[\frac{1}{\varepsilon_m} - \frac{1}{\varepsilon_s}\right] \qquad \text{Eq. S8}$$

where z_i is the valence of ion i, e is the elementary charge, ε_0 is the vacuum permittivity, k_B is the Boltzmann constant, r_i is the cavity radius for ion i, and ε_m and ε_s are the dielectric constants of the polymer and solution, respectively. The subscript 0, introduced in Eq. S8, is provided to differentiate equations stemming from the classic Born model from those stemming from the Freger-Born model that will be differentiated subsequently with a subscript 1. The classic Born model can be used to define the mean ionic excess solvation energy (i.e., the sum of the value described by Eq. S8 for the cation and anion),[3,5] $\Delta W_{s,0}$, as:

$$\Delta W_{s,0} = \frac{e^2}{8\pi\varepsilon_0 k_B T}\left(\frac{z_+^2}{r_+} + \frac{z_-^2}{r_-}\right)\left[\frac{1}{\varepsilon_m} - \frac{1}{\varepsilon_s}\right] \qquad \text{Eq. S9}$$

where z_+ and z_- are the valences of the cation and anion, respectively, and r_+ and r_- are the cavity radii of the cation and anion, respectively. Eq. S9 is useful in that it describes the difference in the mean ionic excess solvation energy (between polymer and solution) for any aqueous electrolyte. For a 1-1 electrolyte (i.e., $z_+ = z_- = 1$), Eq. S9 can be simplified by defining a mean ionic average cavity radius, r_s:

$$\frac{1}{r_s} = \left(\frac{1}{2r_+} + \frac{1}{2r_-}\right) \qquad \text{Eq. S10}$$

The values of r_+ and r_- are taken as the cavity radii of the ions as proposed Duignan et al.[8] The mean ionic cavity radius of NaCl, as defined by Eq. S10, is quantitatively similar to those defined by other applications of the Born model where r_s is defined using the geometric average of the cavity radii of the cation and anion of the salt.[2,9,10] For example, the value of r_s calculated via Eq. S10 is 1.87 Å, and the value of r_s calculated using the geometric average cavity radius is 1.95 Å. This mean ionic average cavity radius is defined so that combination of Eqs. S6, S7, S9, and S10 result in the familiar form of the Electrostatic Theory with the classic Born model for a 1-1 electrolyte:[6,7,9]

$$K_{s,0} = \exp\left[-\frac{e^2}{8\pi\varepsilon_0 k_B T r_s}\left[\frac{1}{\varepsilon_m} - \frac{1}{\varepsilon_s}\right]\right] \quad \text{Eq. S11}$$

The difference in the mean ionic excess solvation energy between polymer and solution described by the Freger-Born model is obtained using a similar procedure as that of the classic Born model. The excess solvation energy for an ion, accounting for the interface between polymer-rich and water-rich regions in the polymer matrix, $\Delta W_{i,1}$, is calculated as:[6]

$$\Delta W_{i,1} = \frac{z_i^2 e^2}{8\pi\varepsilon_0 k_B T r_p}\left[\frac{1}{\varepsilon_m} - \frac{1}{\varepsilon_v}\right] + \frac{z_i^2 e^2}{8\pi\varepsilon_0 k_B T r_i}\left[\frac{1}{\varepsilon_v} - \frac{1}{\varepsilon_s}\right] \quad \text{Eq. S12}$$

where r_p is the characteristic void space of the polymer, which, for a cross-linked hydrogel polymer, can be taken as the network mesh size, and ε_v is the dielectric constant of the water-rich void. Eq. S12 is valid for geometry where $r_p > r_i$.[6] Using the approximation that $\varepsilon_v = \varepsilon_s$,[6] and summing Eq. S12 for the cation and anion, the mean ionic excess solvation energy becomes:

$$\Delta W_{s,1} = \frac{(z_+^2 + z_-^2)e^2}{8\pi\varepsilon_0 k_B T r_p}\left[\frac{1}{\varepsilon_m} - \frac{1}{\varepsilon_s}\right] \quad \text{Eq. S13}$$

Analogous to the derivation of the Electrostatic Theory with the classic Born model (Eq. S11), combining Eqs. S6, S7, and S13 results in the Electrostatic Theory with the Freger-Born model for a 1-1 electrolyte:

$$K_{s,1} = \exp\left[-\frac{e^2}{8\pi\varepsilon_0 k_B T r_p}\left[\frac{1}{\varepsilon_m} - \frac{1}{\varepsilon_s}\right]\right] \quad \text{Eq. S14}$$

The only difference between the Electrostatic Theory with the classic Born model (Eq. S11) and the Electrostatic Theory with the Freger-Born model (Eq. S14) is that, for a 1-1 electrolyte, r_s is replaced by r_p. As such, the Electrostatic Theory with the Freger-Born model is sensitive to polymer chain configuration in a manner that is not captured by the Electrostatic Theory with the classic Born model.

Section S2. Application of the Maxwell Garnett model to hydrated polymers

The Maxwell Garnett model describes the dielectric constants of hydrated polymers experimentally measured via a dielectric relaxation spectroscopy (DRS) technique performed in the microwave frequency range.[11,12] The model (Eq. 3 in the main text) has been applied to a series of cross-linked poly(glycidyl methacrylate) polymers (referred to as XL – pGMA – z) by taking the dielectric constant of pure water as 80 and the dielectric constant of the dry polymer as 2.67 (estimated via a least squares regression).[13] This application of the Maxwell Garnett model, when applied with the polymer taken as the continuous phase, accurately describes the relationship between water content and dielectric constant in hydrated polymers of various structures (e.g. XL – pGMA – z, HEMA/GMA, and sulfonated polysulfone) (Figure S1). Alternatively, this application of the Maxwell Garnett model with water taken as the continuous phase more

accurately describes the dielectric constant of commercially available hydrated Nafion 117 polymers (Figure S1).

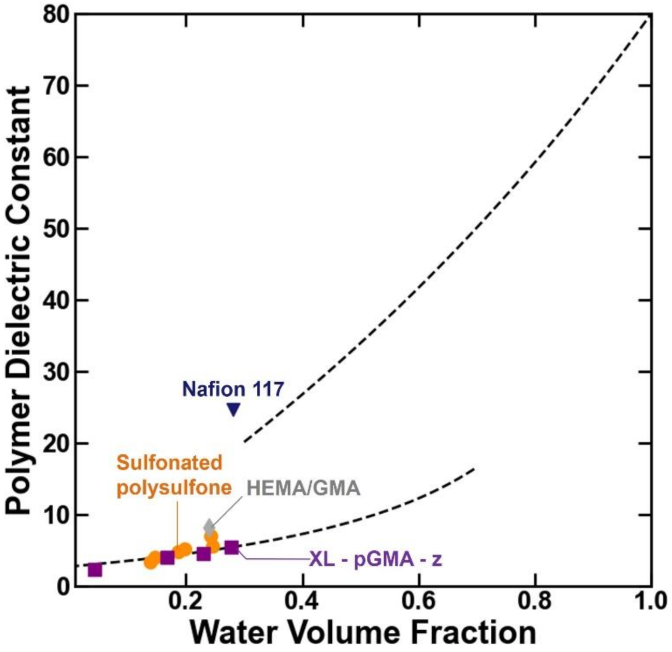

Figure S1. Hydrated polymer dielectric constant plotted as a function of the water volume fraction for HEMA/GMA (♦),[14] sulfonated polysulfone (●),[15] XL – pGMA – z (■),[10] and Nafion 117(▼).[16] The dashed lines were calculated using the Maxwell Garnett Model.

XL – pGMA – z and HEMA/GMA were cross-linked with poly(ethylene glycol) diacrylate so they are structurally similar to XLPEGDA. For this reason, we estimated the dielectric constant of the XLPEGDA using the polymer-continuous application of the Maxwell-Garnett model. For XLPEGDA, the model (Eq. 3 in the main text) was evaluated by taking the dielectric constant of water as 80 and taking the dielectric constant of the dry polymer to be 12 as previously reported.[17]

Calculating the dielectric constant of the hydrated polymers using a polymer-continuous application of the Maxwell Garnett model requires an assumption that the phase transition from polymer to water continuity does not occur in XLPEGDA over water volumes of approximately 0.2 – 0.8 (Figure 1). There is justification for this assumption in the literature because the phase

transition point in heterogeneous phase models, such as the Maxwell Garnett model can be observed at remarkably dilute concentrations of the "continuous" phase. For example, Morisato et al. modeled the nitrogen permeability in a blend of poly(1-trimethylsilyl-1-propyne) (PTMSP) and poly(1-phenyl-1-propyne) (PPP) using the Maxwell model and observed that the continuous phase transition point for PPP or PTMSP continuous applications of the model occurred at a volume fraction of less than 0.1 PPP. [18]

Section S3. Determining the characteristic void space size using the mesh size

The effective mesh size of a cross-linked hydrogel, such as XLPEGDA, can be calculated using the polymer's structural properties when swollen in the presence of a diluent (i.e., water).[19,20] The procedure for this calculation for XLPEGDA hydrogels has been previously described by Ju et al.,[19] but for convenience, the procedure is summarized here. First, the average molecular weight between cross-links, M_c is estimated as:[19]

$$\frac{1}{M_c} = \frac{2}{M_n} - \frac{\frac{v}{V_1}\left[\ln\left(1 - V_{2,s}\right) + V_{2,s} + \chi V_{2,s}^2\right]}{V_2^r\left[\left(\frac{V_{2,s}}{V_{2,r}}\right)^{1/3} - \frac{1}{2}\left(\frac{V_{2,s}}{V_{2,r}}\right)\right]} \qquad \text{Eq. S15}$$

where M_n is the molecular weight of the crosslinker PEGDA, v is the specific volume of the cross-linker (approximated as its value for amorphous poly(ethylene glycol) (PEG), which is 0.893 cm^3/mol), V_1 is the molar volume of the diluent (i.e., water), $V_{2,r}$ is the equilibrium polymer volume fraction after cross-linking but before swelling, $V_{2,s}$ is the polymer volume fraction of the fully swollen hydrogel, and χ is the Flory Huggins interaction parameter, which is 0.426 (approximated as its value for PEG, as is common in similar calculations for PEG-based hydrogels).[19,21–23] Subsequently, the root-mean squared end-to-end distance of PEG chains between cross-links, $(r_0^2)^{1/2}$, is calculated as:[19]

$$\langle r_0^2 \rangle^{1/2} = l\left(\frac{3M_c}{M_r}\right)^{1/2} C_n \qquad \text{Eq. S16}$$

where l is the average bond length (which is 1.50 Å), M_r is the molar mass of the repeat unit (i.e., the molar mass of PEG, which is 44 g/mol) and C_n is the characteristic ratio (approximated as 3.8, which is its value for amorphous PEG). The mesh size, ζ, is then calculated as:[19]

$$\zeta = \langle r_0^2 \rangle^{1/2} V_{2,s}^{-1/3} \qquad \text{Eq. S17}$$

Previously, Ju et al. reported the mesh size (calculated using this approach) for XLPEGDA films equilibrated with de-ionized water.[19] We calculated the mesh size of the XLPEGDA films synthesized by Jang et al. using the same procedure.[24] Sample parameters used in the calculation for Jang et al.'s data are provided for convenience in Table S1.

To synthesize XLPEGDA films over a range of water volume fractions, Jang et al. synthesized polymers from solutions containing pre-polymerization water contents of 0%, 20%, and 40% (Table S1).[24] The polymer nomenclature was such that these films were referred to as XL0, XL20, and XL40, respectively. The mesh size increased with increasing water volume fraction in the polymers, which is generally consistent with scaling relationships observed in other materials.[20,25]

Table S1. Polymer properties used to calculate the mesh size in XL0, XL20, and XL40.[24]

**Material**	**Pre-polymerization water content [%]**[a]	**Water volume fraction, ϕ_w** [a]	V_2^r [b]	V_2^s	**Mesh size, ζ [Å]**
XL0	0	0.37	1	0.63	12
XL20	20	0.44	0.78	0.56	13
XL40	40	0.52	0.57	0.48	15

[a] Reported by Jang et al.[24]
[b] Calculated by assuming volume additivity of polymer and water in the pre-polymerization solution.

For polymers with an equilibrium volume fraction of polymer greater than 0.10, Canal and Peppas suggested that the mesh size can be empirically related to the inverse equilibrium polymer volume fraction in the swollen film.[20] In other words, the mesh size, ζ, scales with the polymer water volume fraction, ϕ_w, according to: $\zeta \sim (1-\phi_w)^{-1}$.[20] We used this scaling law to develop an empirical relationship between the mesh size and polymer water volume fraction in XL0, XL20, and XL40, and the XLPEGDA films synthesized by Ju et al.[19] via a least squares linear regression (Figure S2). The linear equation for the mesh size (shown on Figure S2) provides an avenue to calculate a mesh size based solely on the volume fraction of water in the polymer. This approach may be useful in situations where experimentally determined mesh sizes are not available or practical. For example, $2r_p = \zeta = 5.1(1-\phi_w)^{-1} + 3.4$ was used along with Eq. S14 and Eq. 3 (in the main text) to calculate the solid line in Figure 1 of the main text.

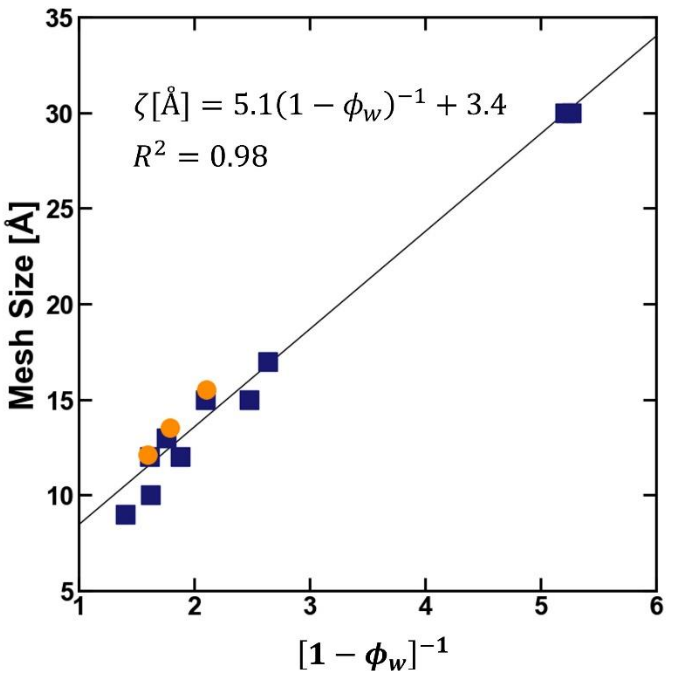

Figure S2. Polymer mesh size reported as a function of the inverse polymer volume fraction (represented here as one minus the water volume fraction). The solid line was calculated by fitting the data using a least-squares linear regression.

Section S4. Determining the solution dielectric constant via experimental correlation models

The concentration dependent dielectric constant of aqueous electrolytes can be modeled using semi-empirical experimental correlation models, which take the general form:[26]

$$\varepsilon_s(C_s^s) = \varepsilon_w + \sum_{i=1}^{5} \theta_i^{EC} {C_s^s}^{i/2} \qquad \text{Eq. S18}$$

where ε_s is the dielectric constant of the solution, C_s^s is the concentration of the external solution, ε_w is the dielectric constant of pure water, i is an arbitrary summation variable, and θ_i^{EC} are empirically determined parameters. The empirically determined parameters are obtained from the literature.[26] The experimental correlation model for NaCl, LiCl, and KCl informed all values of solution dielectric constant for a given application of the Electrostatic Theory.

Table S2. Empirical correlation model parameters, as reported by Silva et al.,[26] used to calculate the concentration dependent dielectric constant of NaCl, KCl, and LiCl solutions via Eq. S18.

Salt	$\boldsymbol{\theta_1^{EC}}$	$\boldsymbol{\theta_2^{EC}}$	$\boldsymbol{\theta_3^{EC}}$	$\boldsymbol{\theta_4^{EC}}$	$\boldsymbol{\theta_5^{EC}}$
NaCl	0	-16.2	3.10	0	0
KCl	0	-14.7	3.00	0	0
LiCl	0	-15.5	0	1.96	-0.306

Section S5. Concentration dependance of LiCl and KCl partitioning in XLPEGDA

Jang et al. characterized the LiCl and KCl partitioning properties of XL0, XL20, and XL40 polymers over a range of 0.01 – 1 M concentrations.[24] Similar to observations for the polymers equilibrated with NaCl, the partition coefficient of polymers equilibrated with KCl and LiCl increases with increasing solution concentration (Figure S3). The Born Model also qualitatively describes this result because the dielectric constant of the external LiCl and KCl solutions decreases as solution concentration increases (Eq. 4 in the main text), however, quantitative agreement between the updated Electrostatic Theory predictions and the experimental data is considerably worse for the polymers equilibrated with KCl and LiCl relative to those equilibrated with NaCl (Table S3). This result is consistent with the physical picture proposed in the main text where associative interactions (e.g., potassium/ethylene oxide association and lithium/water association) influence KCl and LiCl partitioning to a greater extent than NaCl partitioning.

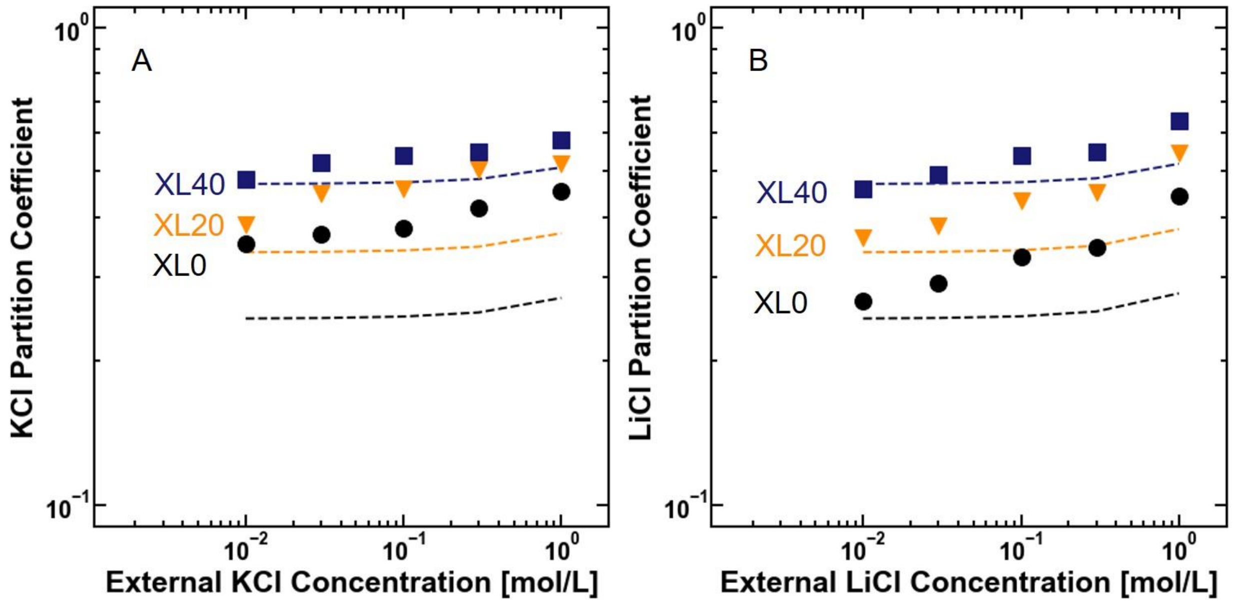

Figure S3. Potassium chloride (A) and lithium chloride (B) partition coefficients for XL0 (●), XL20 (▼), and XL40 (■) reported as a function of external salt concentration. Dashed lines represent predictions made using the Electrostatic Theory calculated with the Freger-Born model.

Table S3. Root mean square (RMS) log error between Electrostatic Theory predictions made with the Classic Born and Born-Freger models and experimental ion sorption data for XL0, XL20, and XL40. RMS log errors were calculated for each polymer/salt over the full range of external concentrations (0.01 M – 1 M) as described previously.[2]

Material	Model	RMS log error		
		NaCl	LiCl	KCl
XL0	Born-Freger	0.05	0.16	0.23
	Classic Born	1.31	1.74	1.30
XL20	Born-Freger	0.02	0.13	0.15
	Classic Born	1.12	1.49	1.09
XL40	Born-Freger	0.04	0.07	0.07
	Classic Born	0.89	1.20	0.85

Section S6. References

(1) Helferrich, F. *Ion Exchange*; McGraw-Hill: New York, 1962.

(2) Bannon, S. M.; Geise, G. M. Influence of Donnan and Dielectric Exclusion on Ion Sorption in Sulfonated Polysulfones. *Journal of Membrane Science* **2024**, *694*, 122396. https://doi.org/10.1016/j.memsci.2023.122396.

(3) Pitzer, K. S. *Activity Coefficients in Electrolyte Solutions*, 2nd ed.; CRC Press: Boca Raton, 1991.

(4) Geise, G. M.; Paul, D. R.; Freeman, B. D. Fundamental Water and Salt Transport Properties of Polymeric Materials. *Progress in Polymer Science* **2014**, *39* (1), 1–42. https://doi.org/10.1016/j.progpolymsci.2013.07.001.

(5) Atkins, P. W.; De Paula, J. *Atkins' Physical Chemistry*, 10th ed.; Oxford University Press: Oxford, New York, 2014.

(6) Freger, V. Dielectric Exclusion, an Éminence Grise. *Advances in Colloid and Interface Science* **2023**, *Volume 319*, 102972. https://doi.org/10.1016/j.cis.2023.102972.

(7) Yaroshchuk, A.; Bruening, M. L.; Zholkovskiy, E. Modelling Nanofiltration of Electrolyte Solutions. *Advances in Colloid and Interface Science* **2019**, *268*, 39–63. https://doi.org/10.1016/j.cis.2019.03.004.

(8) Duignan, T. T.; Parsons, D. F.; Ninham, B. W. A Continuum Solvent Model of the Partial Molar Volumes and Entropies of Ionic Solvation. *J. Phys. Chem. B* **2014**, *118* (11), 3122–3132. https://doi.org/10.1021/jp410956m.

(9) Zhang, H.; Geise, G. M. Modeling the Water Permeability and Water/Salt Selectivity Tradeoff in Polymer Membranes. *Journal of Membrane Science* **2016**, *520*, 790–800. https://doi.org/10.1016/j.memsci.2016.08.035.

(10) Chang, K.; Luo, H.; Geise, G. M. Water Content, Relative Permittivity, and Ion Sorption Properties of Polymers for Membrane Desalination. *Journal of Membrane Science* **2019**, *574*, 24–32. https://doi.org/10.1016/j.memsci.2018.12.048.

(11) Chang, K.; Geise, G. M. Dielectric Permittivity Properties of Hydrated Polymers: Measurement and Connection to Ion Transport Properties. *Ind. Eng. Chem. Res.* **2020**, *59* (12), 5205–5217. https://doi.org/10.1021/acs.iecr.9b03950.

(12) Lou, J.; Hatton, T. A.; Laibinis, P. E. Effective Dielectric Properties of Solvent Mixtures at Microwave Frequencies. *J. Phys. Chem. A* **1997**, *101* (29), 5262–5268. https://doi.org/10.1021/jp970731u.

(13) Chang, K.; Luo, H. Influence of Salt Concentration on Hydrated Polymer Relative Permittivity and State of Water Properties. *Macromolecules* **2021**, *54* (2), 637–646. https://doi.org/10.1021/acs.macromol.0c02188.

(14) Luo, H.; Chang, K.; Bahati, K.; Geise, G. M. Functional Group Configuration Influences Salt Transport in Desalination Membrane Materials. *Journal of Membrane Science* **2019**, *590*, 117295. https://doi.org/10.1016/j.memsci.2019.117295.

(15) Chang, K.; Luo, H.; Lin, S. Y.; Agata, W.-A. S.; Geise, G. M.; Sean M. Bannon. Methoxy Groups Increase Water and Decrease Salt Permeability Properties of Sulfonated Polysulfone Desalination Membranes. *Journal of Membrane Science* **2021**, *630*, 119298. https://doi.org/10.1016/j.memsci.2021.119298.

(16) Lu, Z.; Polizos, G.; Macdonald, D. D.; Manias, E. State of Water in Perfluorosulfonic Ionomer (Nafion 117) Proton Exchange Membranes. *Journal of The Electrochemical Society* **2008**, *155* (2), 10. https://doi.org/10.1149/1.2815444.

(17) Kalakkunnath, S.; Kalika, D. S.; Lin, H.; Raharjo, R. D.; Freeman, B. D. Molecular Relaxation in Cross-Linked Poly(Ethylene Glycol) and Poly(Propylene Glycol) Diacrylate Networks by Dielectric Spectroscopy. *Polymer* **2007**, *48* (2), 579–589. https://doi.org/10.1016/j.polymer.2006.11.046.
(18) Morisato, A.; Shen, H. C.; Sankar, S. S.; Freeman, B. D.; Pinnau, I.; Casillas, C. G. Polymer Characterization and Gas Permeability of Poly(1-Trimethylsilyl-1-Propyne) [PTMSP], Poly(1-Phenyl-1-Propyne) [PPP], and PTMSP/PPP Blends. *Journal of Polymer Science Part B: Polymer Physics* **1996**, *34* (13), 2209–2222. https://doi.org/10.1002/(SICI)1099-0488(19960930)34:13<2209::AID-POLB10>3.0.CO;2-9.
(19) Ju, H.; McCloskey, B. D.; Sagle, A. C.; Kusuma, V. A.; Freeman, B. D. Preparation and Characterization of Crosslinked Poly(Ethylene Glycol) Diacrylate Hydrogels as Fouling-Resistant Membrane Coating Materials. *Journal of Membrane Science* **2009**, *330* (1–2), 180–188. https://doi.org/10.1016/j.memsci.2008.12.054.
(20) Canal, T.; Peppas, N. A. Correlation between Mesh Size and Equilibrium Degree of Swelling of Polymeric Networks. *Journal of Biomedical Materials Research* **1989**, *23* (10), 1183–1193. https://doi.org/10.1002/jbm.820231007.
(21) Della Sala, F.; Biondi, M.; Guarnieri, D.; Borzacchiello, A.; Ambrosio, L.; Mayol, L. Mechanical Behavior of Bioactive Poly(Ethylene Glycol) Diacrylate Matrices for Biomedical Application. *Journal of the Mechanical Behavior of Biomedical Materials* **2020**, *110*, 103885. https://doi.org/10.1016/j.jmbbm.2020.103885.
(22) Cima, L. G.; Lopina, S. T. Network Structures of Radiation-Crosslinked Star Polymer Gels. *Macromolecules* **1995**, *28* (20), 6787–6794. https://doi.org/10.1021/ma00124a013.
(23) Nguyen, Q. T.; Hwang, Y.; Chen, A. C.; Varghese, S.; Sah, R. L. Cartilage-like Mechanical Properties of Poly (Ethylene Glycol)-Diacrylate Hydrogels. *Biomaterials* **2012**, *33* (28), 6682–6690. https://doi.org/10.1016/j.biomaterials.2012.06.005.
(24) Jang, E.-S.; Kamcev, J.; Kobayashi, K.; Yan, N.; Sujanani, R.; Dilenschneider, T. J.; Park, H. B.; Paul, D. R.; Freeman, B. D. Influence of Water Content on Alkali Metal Chloride Transport in Cross-Linked Poly(Ethylene Glycol) Diacrylate.1. Ion Sorption. *Polymer* **2019**, *178*, 121554. https://doi.org/10.1016/j.polymer.2019.121554.
(25) Richbourg, N. R.; Peppas, N. A. The Swollen Polymer Network Hypothesis: Quantitative Models of Hydrogel Swelling, Stiffness, and Solute Transport. *Progress in Polymer Science* **2020**, *105*, 101243. https://doi.org/10.1016/j.progpolymsci.2020.101243.
(26) Silva, G. M.; Liang, X.; Kontogeorgis, G. M. How to Account for the Concentration Dependency of Relative Permittivity in the Debye–Hückel and Born Equations. *Fluid Phase Equilibria* **2023**, *566*, 113671. https://doi.org/10.1016/j.fluid.2022.113671.
